# Unraveling Spatial Patterns and Drivers of Fish Ecological Uniqueness in Subtropical Streams

**DOI:** 10.1002/ece3.71112

**Published:** 2025-03-27

**Authors:** Jialing Qiao, Ling Chu, Yuru Li, Tianjiang Chu, Nan Xie, Yunzhi Yan

**Affiliations:** ^1^ Institute of Fishery Science Hangzhou Academy of Agricultural Sciences Hangzhou China; ^2^ Institute of Hydrobiology Chinese Academy of Sciences Wuhan China; ^3^ School of Ecology and Environment Anhui Normal University Wuhu China; ^4^ School of Fisheries Zhejiang Ocean University Zhoushan China

**Keywords:** functional traits of fish, land use, local contribution to β‐diversity, species contribution to β‐diversity, β‐diversity

## Abstract

β‐diversity is effective for measuring the degree of compositional variation among communities and can be decomposed into local (LCBD) and species (SCBD) contributions to β‐diversity. Previous studies on β‐diversity have mostly been limited to the taxonomic level; relatively few studies have been conducted on functional diversity and its two components based on species functional traits, which have seriously hindered the potential application of β‐diversity in conservation biology. In addition, increasing anthropogenic disturbance has led to uncertainties in the β‐diversity of fish communities in stream ecosystems. In this study, we explored the relationships between multidimensional β‐diversity of fish communities and regional landscape factors, local environmental factors, community metrics, and species functional traits to reveal the main drivers of β‐diversity in headwater streams of the Xin'an River, China. LCBD and SCBD values were calculated based on species abundance and functional traits, that is, local and species contribution to taxonomic (TLCBD and TSCBD, respectively) and functional (FLCBD and FSCBD, respectively) β‐diversity. The results showed that LCBD and SCBD values were affected by biological and environmental factors. Among biological drivers, TLCBD values were positively related to species abundance. FLCBD values were positively related to species abundance, functional originality, specialization, and dispersion, that is, sampling sites with greater ecological uniqueness at the taxonomic level also had greater ecological uniqueness at the functional level. Among environmental drivers, TLCBD and FLCBD values were negatively related to water temperature, substrate coarseness, and agricultural and urban land use, that is, anthropogenic land use and local environmental changes reduce the ecological uniqueness of fish communities in the studied streams. For SCBD, both TSCBD and FSCBD values were affected by species abundance, occupancy, and niche position. We suggest that consideration of SCBD as part of a conservation approach needs to be coupled with species abundance and occupancy, which will help to prioritize areas for conservation and restoration in the study region.

## Abstract 2

Our results revealed differences in the spatial distribution patterns of β‐diversity and its drivers, indicating the importance of the decomposition of β‐diversity into local (LCBD) and species (SCBD) contributions to β‐diversity. The conversion of natural landscapes into artificial landscapes around streams has decreased the ecological uniqueness (TLCBD) of fish communities in the headwater stream of the Xin'an River, China. Our study suggests that conservation efforts should consider both LCBD and SCBD in contexts and emphasize the importance of protecting headwater streams.

## 1 Introduction

Understanding biodiversity dynamics and its driving factors is a key area of focus in conservation biology (Socolar et al. 2016). Among the many measures of biodiversity, β‐diversity can measure compositional differences between communities and be used to explore the distribution patterns and driving mechanisms of communities along environmental gradients (Whittaker 1972; Mori et al. 2018). The traditional method of calculating β‐diversity is to treat all species within a community as the same functional roles, and studies are limited to the taxonomic level (i.e., taxonomic β‐diversity; Whittaker 1972). In recent years, with the development of functional ecology, an increasing number of researchers have found that due to interspecific differences in functional traits, species differ in their ecological status in the community and their responses to environmental changes (Devictor et al. 2010; Jiang et al. 2021). Many studies have also reported that functional diversity, based on functional traits of different species—that is, morphophysiological and behavioral characteristics—can compensate for deficiencies in taxonomic diversity, particularly in revealing mechanisms of community assembly (Jiang et al. 2021; Qiao et al. 2022; Brandl et al. 2023). In addition, since functional traits mediate the effects of environmental factors on communities, the functional traits of species determine species–environment relationships (Brumm et al. 2023). Thus, functional β‐diversity is thought to provide valuable insights into community responses to anthropogenic disturbances (Bergholz et al. 2017).

In recent years, several researchers have decomposed the total dissimilarity among communities within a region (total β‐diversity, BDTotal) into local contribution to β‐diversity (LCBD) and species contribution to β‐diversity (SCBD) to determine which sites and species should be prioritized for protection in anthropogenically disturbed ecosystems (Legendre and De Caceres 2013). LCBD is ecological uniqueness at the community level, which represents the degree of ecological uniqueness of the local community compared to that of other communities within the studied area. Higher LCBD values indicate that the community has a different species composition from other communities. For instance, Heino et al. (2017) reported that sites with high LCBD values within degraded landscapes are preserved sites harboring sensitive and rare species. SCBD is ecological uniqueness at the species level, which represents the degree of ecological uniqueness of the single species compared to that of other species within the studied area. A higher SCBD value indicates that this species contributes more to β‐diversity than other species in the studied area. LCBD and SCBD have recently become popular topics in terrestrial and freshwater research due to their potential role in diversity conservation and restoration planning (Heino et al. 2017; Vilmi et al. 2017; da Silva et al. 2018; Wang et al. 2022). However, SCBD and its ecological correlates have received relatively little attention to date compared with the considerable scholarly focus on LCBD (Heino and Gronroos 2017; da Silva et al. 2018).

SCBD values are associated with several intrinsic characteristics of species, including species occupancy, species abundance, functional traits, niche position (i.e., the extent to which the environmental conditions in which a species occurs are typical of all available conditions), and niche breadth (i.e., the range of environmental conditions in which a species occurs) (Heino and Gronroos 2017; da Silva et al. 2018). For instance, if the abundance of a single species at different sites varies greatly, this will increase β‐diversity (Heino and Gronroos 2017). The species that are widely distributed among sites also tend to increase in diversity (Heino and Gronroos 2017). These changes in the abundance and distribution of species have a significant impact on β‐diversity values.

The magnitude of LCBD and SCBD values is influenced by both biological and environmental factors (Heino and Gronroos 2017; da Silva et al. 2018). Studying their response to environmental changes can reveal the compositional characteristics of species and the direction of community succession. For instance, Borges et al. (2020) reported that the LCBD values in fish communities increased with greater anthropogenic disturbance in the upper Parana River. The study also revealed that the fish communities in the studied area were dominated by species that were less specialized and more tolerant to environmental changes. In addition, trade‐offs between biological factors and ecological uniqueness can be considered in conservation planning by studying the relationships between LCBD values and biological factors (Heino et al. 2017; Vilmi et al. 2017; Xia et al. 2022; Wang et al. 2022). For instance, several researchers have shown that many species with high ecological differentiation are present in areas where “LCBD values are positively correlated with taxonomic α‐diversity” (Beisel et al. 2000; Legendre and De Caceres 2013; Kong et al. 2017; Pajunen et al. 2017). Such scenarios tend to occur in streams with low levels of anthropogenic disturbance and in areas with relatively abundant microhabitats and resources (Miserendino and Masi 2010; Jun et al. 2011). Qiao et al. (2015) reported that common species had a greater negative impact on the correlation between LCBD and taxonomic α‐diversity. LCBD values were also found to be related to functional α‐diversity (Xia et al. 2022). In the Atlantic Forest, da Silva et al. (2020) consistently observed a negative association between high LCBD values in dung beetles and functional α‐diversity, LCBD values in mammals did not show a similar correlation with functional diversity. However, it is currently uncertain how changes in the LCBD values of fish communities in stream ecosystems are explained by functional α‐diversity.

Headwater streams play a vital role in the river network (Carvalho et al. 2021). In recent decades, the conversion of natural landscapes to artificial landscapes around streams has severely impacted the species composition and diversity of biological communities (Fierro et al. 2017; Reid et al. 2018; Morrison et al. 2020; Petsch et al. 2021). For instance, Liu et al. (2024) used macroinvertebrates as study subjects and determined that 35%–50% of the sampling sites in riverine tributaries of Thousand Islands Lake could preserve about 60% of the area's ecological uniqueness. However, the factors influencing the ecological uniqueness of stream fishes and their response to human disturbance gradients in stream ecosystems have been relatively understudied (Schneck et al. 2022; Xia et al. 2022). This study aimed to shed light on this topic (Bojsen and Barriga 2002; Göthe et al. 2015; Edge et al. 2017; Erős et al. 2020). This research evaluated the patterns of LCBD and SCBD, and their driving factors, in headwater streams fish communities along the gradient of land‐use intensification (from forested streams to streams surrounded by anthropic land use) in the Xin'an River, China. The main purpose of this study was to explore four aspects: (1) What are the spatial patterns of fish ecological uniqueness in the studied area?; (2) the LCBD values of fish communities were affected by biological (e.g., community abundance and functional α‐diversity) and environmental factors (e.g., local and regional scale environmental variables); (3) stream sites affected by more intense anthropogenic land use have a lower LCBD; and (4) the SCBD values were affected by biological variables (e.g., total abundance and occupancy, niche position) of fish communities.

## 2 Materials and Methods

### 2.1 Study Area

The Xin'an River is one of the major rivers in the Wannan mountainous China, which belongs to the Qiantang River system. It originates from the northern foot of Wugujian in southwestern Xiuning County, Anhui Province, and flows from west to east through Xiuning County, Tunxi District, Shexian County, Huangshan city, and Jiekou town to join Qiandao Lake in Zhejiang Province. In Anhui Province, the mainstream length of the Xin'an River is approximately 240 km, and the basin area is approximately 6440 km^2^, accounting for nearly 55.7% of the total basin area. Since Anhui Province has a subtropical monsoon climate, the air temperature and precipitation in the Xin'an River exhibit seasonal changes. In the past 30 years, the average monthly temperature has ranged from 4.2°C (January) to 27.9°C (July), and the annual rainfall is approximately 1800 mm/y, of which close to 80% falls from April to September.

### 2.2 Field Sampling

**FIGURE 1:**
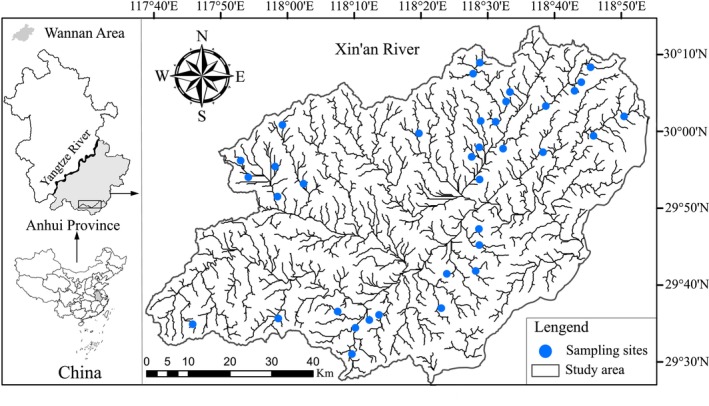
Sampling sites in the Xin'an River of Wannan mountains, China.

To collect fish specimens, 36 sampling sites were established along the land‐use gradient of the Xin'an River in August 2012 (Figure 1). A backpack electrofishing device (CWB‐2000 P, China; 12‐V import, 250‐V export) was used to capture specimens from each sampling site, with one person responsible for electrofishing and two people for catching fish with a net (approximately 30 min of sampling time per 50‐m sampling segment). Sampling sites were selected based on habitat accessibility and representativeness and to cover as many microhabitats in the segment as possible, especially the most typical and common riffles and pools. The collected specimens were identified and counted in the field. Afterwards, the remaining live specimens were immediately released back to the sampling site, and the nonlive specimens were preserved in 8% formalin and taken to the laboratory for further analyses (L. Lin et al. 2021).

### 2.3 Local and Landscape Scale Environmental Variables

To characterize the habitat at each sampling site, nine local environmental variables, including water temperature (°C), water depth (m), water width (m), current velocity (m/s), flow (m^3^/s), conductivity (μs/cm), dissolved oxygen (mg/L), substrate coarseness, and heterogeneity, were measured during fish sampling. Wetted width (WW) was measured at five equally spaced transects along each stream channel. Water depth (WD) was measured at five equally spaced points along each transect, and current velocity (CV) was measured at 60% water depth at each equidistant point (FP111, USA). The flow (FL) at each site was obtained by multiplying the mean WW, WD, and CV. Water temperature (WT), conductivity (CO) and dissolved oxygen (DO) were measured at each site using a YSI ProPlus (Yellow Springs Instruments). Following Bain et al. (1985) substrate type classification method, each sampled segment was divided into 10 transects, and the substrate type was estimated by visually measuring the diameter of the substrate structure at each transect. Since the substrate was composed of different sized structures, it was divided into six categories: (i) 0: < 0.06 mm; (ii): 0.06–1 mm; (iii): 2–15 mm; (iv): 16–63 mm; (v): 64–256 mm; and (vi): > 256 mm. The mean of the substrate coarseness is the substrate roughness (SC) and the standard deviation is the substrate heterogeneity (SH).

To estimate the impact of landscape scale variables on LCBD values better, the land use around the sampling sites (500‐m radius) was quantified to indicate the landscape scale variables (Barbosa et al. 2020). The data were extracted using a combination of the digital elevation model and the land use dataset of Anhui Province through ArcGIS 10.8. The land‐use dataset was provided by the Data Center for Resources and Environmental Sciences, Chinese Academy of Science (http://www.redc.cn). Within each area, five land‐use types were categorized as follows: (1) agricultural land (AL): paddy fields and nonirrigated farmland; (2) forest land (FA): macrophanerophytes, shrubs, and bamboo; (3) grass land (GL): herbage; (4) Water body (WB): rivers, canals, lakes and reservoirs, and ponds; and (5) urban land (UL): paved areas, cement pavement, residences, industries, and other construction land. Since the effects of land use on habitat conditions and aquatic organisms are time delayed (Camana et al. 2020), this study also extracted the land‐use dataset from 1980 for each site. The rate of area change (m^2^/y) of agricultural land (RAL), forest land (RFA), grass land (RGL), water body (RWB), and urban land (RUL) from 1980 to 2010 was obtained at each site for further analysis. The basic information on the local environmental and regional variables was presented in Table 1.

**TABLE 1:** Basic information on local environmental variables and regional land‐use variables in the study area.

Environmental variables	Names	Abbreviation	Range	Mean ± SD
Local‐scale environmental variables	Dissolved oxygen (mg/L)	DO	2.72–12.49	7.74 ± 1.97
	Water temperature (°C)	WT	18.8–33.8	26.56 ± 4.71
	Conductivity (μS/cm)	CO	18.28–275.2	121.08 ± 73.02
	Water width (m)	WW	2.6–120	16.74 ± 23.4
	Water depth (m)	WD	0.07–1.01	0.32 ± 0.24
	Current velocity (m/s)	CV	0.07–0.57	0.21 ± 0.12
	Flow (m^3^/s)	FL	0.03–14.44	1.3 ± 2.77
	Substrate coarseness	SC	1.3–5.8	3.36 ± 1.08
	Substrate heterogeneity	SH	0.3–1.69	0.96 ± 0.39
Land‐use variables	Agricultural land (%)	AL	2.71–98.08	44.6 ± 29.92
	Forest land (%)	FA	0–97.29	48.3 ± 32.77
	Grass land (%)	GL	0–54.91	2.19 ± 9.75
	Water body (%)	WB	0–20.55	1.53 ± 4.71
	Urban land (%)	UL	0–24.45	3.38 ± 6.11
	Agricultural land (m^2^/y)	RAL	−117.74 to 4556.39	482.78 ± 1238.22
	Forest land (m^2^/y)	RFA	−4544.43 to 117.74	−129.68 ± 758.87
	Grass land (m^2^/y)	RGL	−19.68 to 42.08	0.62 ± 7.83
	Water body (m^2^/y)	RWB	−114.45 to 60	0.33 ± 23.25
	Urban land (m^2^/y)	RUL	−3163.18 to 60	−103.51 ± 534.29

### 2.4 Functional Traits

To calculate functional diversity, 15 morphological and ecological traits were selected. These traits fulfill the following two criteria: (1) they are related to fish functions such as locomotion, habitat use, food acquisition, and reproduction; and (2) they can be used to capture fish responses to environmental changes (Violle et al. 2007; F. Lin et al. 2021). The morphological traits consisted of 13 continuous traits related to locomotion/habitat use, namely, body elongation, body transverse shape, caudal peduncle throttling, compression index, body transverse surface, and pectoral fin position. The food acquisition traits are related to eye position, oral gape position, eye size, and gut length. The locomotion/food acquisition traits included body length and body mass. The ecological traits included two categorical variables, namely, habitat type, and water column. In this study, at least 20 specimens of each species were measured for functional traits in the laboratory. If the minimum sample size was < 20 specimens, we measured all the individuals. The detailed methods for measuring and processing morphological and ecological traits were consistent with our previous work (Qiao et al. 2024). We assumed that interspecific variation in traits was greater than intraspecific variation, and we used the average trait ratio and categorical variables of each species based on adults to quantify functional diversity.

### 2.5 Data Analysis

#### 2.5.1 Measurement of Total β‐Diversity (BD_total_ ), LCBD, and SCBD

Taxonomic total β‐diversity (TBD_total_) and its two components, local contribution to taxonomic β‐diversity (TLCBD) for each site and species contribution to taxonomic β‐diversity (TSCBD) for each species, were calculated based on Hellinger‐transformed abundance data (Legendre and De Caceres 2013). Similarly, species abundance and functional trait data were combined to calculate the total functional β‐diversity (FBD_total_), local contribution to functional β‐diversity (FLCBD) for each site, and species contribution to functional β‐diversity (FSCBD) for each species (da Silva et al. 2018, 2020). Since the functional traits of the species were both continuous and categorical, prior to calculating functional β‐diversity, we calculated the trait distance matrix between species based on the Gower distance, which allows different types of variables to be combined while giving them equal weight (Gower 1971; de Bello et al. 2021). The BD_total_, LCBD, and SCBD were calculated in R 3.6.3. Details can be found in the R script posted on GitHub (https://github.com/GabrielNakamura/BetaDiv_extension). The TLCBD and TSCBD were calculated using the “beta.div” function of the “adespatial” package (Dray et al. 2023). The Gower distance was quantified using the “gawdis” function of the “gawdis” package (de Bello et al. 2022). The FLCBD and FSCBD were calculated by the “Beta.div_adapt” function (Nakamura et al. 2020).

#### 2.5.2 Measurement of Function Attributes Information

To explore whether functional α‐diversity affects LCBD, according to the multidimensional framework of Mouillot et al. (2013), six functional α‐diversity indices, namely, functional richness (Fric), functional evenness (Feve), functional divergence (Fdiv), functional dispersion (Fdis), functional specialization (Fspe), and functional originality (Fori), were quantified. In this study, functional imbalance (Fimb) was adopted to estimate functional structure because functional evenness cannot describe the regularity of species abundance in a constructed functional space (Ricotta et al. 2022). A principal coordinate analysis (PCoA) was conducted using the Gower distance matrix, and the first two axes were retained to calculate the functional α‐diversity index. The functional imbalance was calculated using the “FunImbalance” function of the “adiv” package (Pavoine 2020), and the other five indices were quantified using the “alpha.fd.multidim” function of the “mFD” package (Magneville et al. 2022).

#### 2.5.3 Measurement of Community Attributes Information

To estimate whether community attributes affect LCBD and SCBD, community metrics (the frequency of occurrence of species [FO], relative abundance [RA], and species abundance), several niche (niche position and niche breadth), and functional characteristics (functional uniqueness (FUI)) were quantified. The FO and RA of species were calculated in this study. According to FO, species with FO ≥ 40%, 10% ≤ FO < 40%, and FO < 10% were regarded as common, accidental, and rare species, respectively (Chu et al. 2015). To eliminate the effects of extreme values, species abundance was log(*x* + 1) transformed. We used the outlying mean index (OMI) and tolerance index (TOL) to determine the niche position and niche breadth, respectively, for each species (Dolédec et al. 2000). This approach estimates species marginality, which refers to the distance between the mean environmental conditions used by a species and the mean environmental conditions available within the study area. Species with high OMI values tend to have marginal niches, and those with low OMI values have nonmarginal niches. This method also provides a variance term called “tolerance” (TOL; Tales et al. 2004), which quantifies the range for the distribution of each fish species along environmental gradients. High TOL values indicate that species have wide niches, whereas low TOL values indicate narrow niches. According to Heino and Grönroos (2014), all species and environmental variables (including local environmental variables and regional landscape variables) were used to calculate niche position and niche breadth in this study. Since the measure of species niche characteristics tends to include very rare species, such as those occurring at only one site, the TOL estimate may be zero. Although it is virtually impossible for a null niche to exist, the TOL calculation of a zero‐value species is useful for comparison with that of other species because such a zero value is more often represented by species with very small niche widths in the study area (Heino and Grönroos 2014). The FUI value quantifies the degree to which a given species is not functionally equivalent to other species in the entire study area. FUI was calculated using Gower's distance matrix and Hellinger‐transformed species abundance data. OMI and TOL were calculated by the “niche” function of the “ade4” package (Dray and Dufour 2007); FUI was quantified using the “uniqueness” function of the “funrar” package (Grenié et al. 2017).

#### 2.5.4 Measurement of Local and Regional Scale Environmental Variables

To better assess whether environmental variables affect the LCBD, all local environmental variables were log(*x* + 1)‐transformed, the percentage of regional landscape area was arcsine(*x*)‐transformed, and the change rate of the regional landscape area was log(*x* + 1)‐transformed to meet the assumptions of multivariate normality (Hinkley and Runger 1984). Since spearman correlation analyses determined that the correlation coefficients between FL and CV, and between AL and FA were both > 0.7, CV and AL were retained for further analysis because they contain additional ecological information (Dormann et al. 2013).

#### 2.5.5 Statistical Analysis

To better evaluate the biological and environmental drivers (community attributes, function attributes, and environmental variables) of LCBD and SCBD values, beta regression analyses were used to assess the relationship between LCBD and SCBD values and their drivers (Heino and Gronroos 2017; Santos et al. 2021; Schneck et al. 2022; Xia et al. 2022). We chose beta regression as our modeling tool because the beta regression method is naturally heteroskedastic and easily accommodates asymmetries; it is appropriate for empirical studies with LCBD and SCBD values varying from 0 to 1 (Cribari‐Neto and Zeileis 2010; Xia et al. 2022). In addition, beta regression is based on the assumptions that the LCBD and SCBD values are beta‐distributed and that they are related to a set of regressors by a linear or nonlinear predictor with unknown coefficients and a link function (Cribari‐Neto and Zeileis 2010). The pseudo coefficient of determination (pseudo R^2^) of the model can be used to evaluate the explanatory power of the overall model for the corresponding variable changes and to test the significance of each explanatory variable in the model to determine the key factors that have a significant effect on the LCBD and SCBD values. Before analysis, all variables (biological and environmental variable) were transformed to better fit the beta regression model (Landeiro et al. 2018; da Silva et al. 2020). Beta regression was quantified using the “betareg” function of the “betareg” package (Cribari‐Neto and Zeileis 2010). The regression curve was plotted using the “ggplot” function of the ggplot2 package (Wickham 2011).

## 3 Results

### 3.1 Species Composition

In total, 4049 specimens comprising 29 species belonging to five orders and 10 families of fishes were collected in this study. Among them, Cypriniformes was the most abundant, accounting for 75.86% of the total species. *Zacco platypus* was the most common (FO%: 94.44%) and most abundant (RA%: 24.65%) species. *Rhinogobius* spp. and * Vanmanenia stenosoma* were the next most common species, with FO% and RA% of 83.33% and 20.67%, 55.56% and 14.87%, respectively. Species such as *Leptobotia taeniops* , *Rhynchocypris oxycephalus*, and *Gnathopogon imberbis* occurred very rarely and in small numbers (FO% < 10%, RA% < 10%; Table S1).

### 3.2 Spatial Patterns of TLCBD and FLCBD and Their Drivers

**FIGURE 2:**
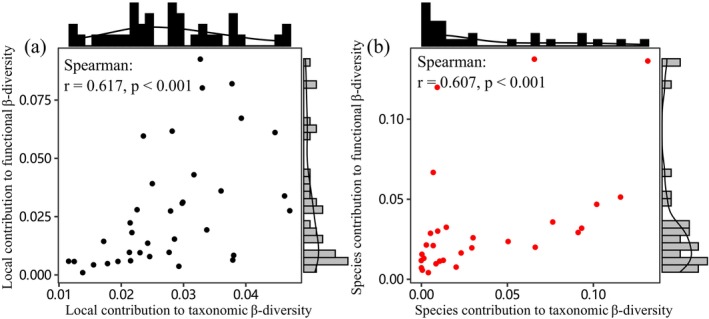
Correlations between taxonomic and functional β‐diversity. (a) Local contribution to taxonomic and functional β‐diversity; (b) species contribution to taxonomic and functional β‐diversity.

The pattern of fish ecological uniqueness at the community level in the study area was as follows: TLCBD values ranged from 0.0117 to 0.0472, and the TLCBD values of 19 sampling sites were higher than the average value of all sampling sites; FLCBD values ranged from 0.0012 to 0.0927, with 14 sampling sites having values higher than the average value of all sampling sites (Table S2). Spearman correlation analysis revealed a moderate positive correlation between TLCBD and FLCBD (*r* = 0.617, *p* < 0.001; Figure 2a).

**FIGURE 3:**
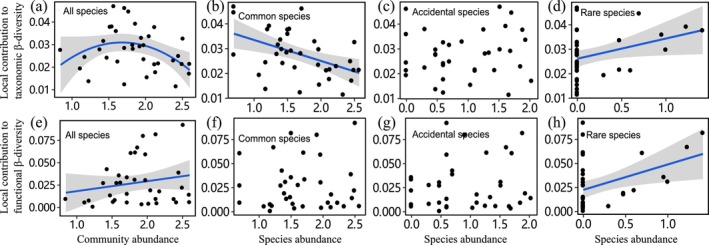
Relationships were fitted between LCBD values (taxonomy and function) and community metrics (abundance of all species, common species, accidental species, and rare species) separately. Shaded gray areas represent the confidence interval of 95% for the regression model. (a)‐(d) Represented the relationship between the local contribution to taxonomic β‐diversity and the abundance of all species, common species, accidental species, and rare species, respectively; (e)‐(h) represented the relationship between the local contribution to functional β‐diversity and the abundance of all species, common species, accidental species, and rare species, respectively.

**FIGURE 4:**
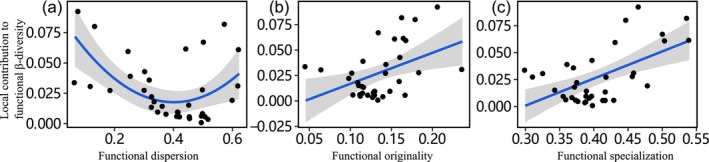
Relationships were fitted between FLCBD values and function metrics separately. Only statistically significant (*p* < 0.05) relationships were showed in here. Shaded gray areas represent the confidence interval of 95% for the regression model. (a)‐(c) Represented the relationship between the local contribution to functional β‐diversity and functional dispersion, functional originality, and functional specialization, respectively.

In the present study, LCBD values of stream fish communities were shown to be affected by biological and environmental factors. Regarding biological drivers, Beta regression analyses revealed that the TLCBD value showed a significant hump‐shaped relationship with community abundance (Table 2; Figure 3a), where the TLCBD value was significantly negatively correlated with the abundance of common species and positively correlated with the abundance of rare species (Table S3; Figure 3b,d). The FLCBD value was positively associated with community abundance (Table 3; Figure 3e), where we found that only the FLCBD value was significantly positively related to rare species abundance (Table S3; Figure 3h). Regarding functional α‐diversity, the FLCBD value was positively linearly correlated with functional originality and functional specialization and a U‐shaped association to functional dispersion (Table 3; Figure 4a–c).

**TABLE 2:** Descriptive statistics of beta regression analyses when community metrics, local‐scale environmental variables and regional land‐use variables were used as predictors of fish TLCBD.

	Estimate	SE	*z*	*p*	Pseudo‐*R* ^2^
Community metrics					
Intercept	−3.583	1.492	−2.401	**0.016**	0.173
Species richness	−3.323	2.966	−1.120	0.263	
Species richness^2^	1.704	1.601	1.064	0.287	
Community abundance	1.943	0.903	2.152	**0.031**	
Community abundance^2^	−0.558	0.242	−2.307	**0.021**	
Local‐scale environmental variables					
Intercept	−1.433	1.104	−1.298	0.194	0.387
DO	−0.642	0.446	−1.439	0.150	
WT	−1.973	0.709	−2.782	**0.005**	
CO	0.075	0.193	0.391	0.696	
WW	0.145	0.177	0.822	0.411	
WD	1.322	0.946	1.397	0.162	
CV	1.529	1.155	1.324	0.185	
SC	1.246	0.510	2.444	**0.015**	
SH	−0.268	0.790	−0.339	0.735	
Land‐use variables					
Intercept	−3.356	0.085	−39.325	**< 0.001**	0.244
AL	−0.399	0.149	−2.684	**0.007**	
GL	−0.293	0.506	−0.579	0.562	
WB	−1.008	1.178	−0.856	0.392	
UL	0.395	0.874	0.452	0.651	
Intercept	−3.58E+00	5.40E−02	−66.298	**< 0.001**	0.300
RAL	3.70E+01	1.16E+02	0.319	0.750	
RFA	5.40E+02	4.25E+02	1.269	0.205	
RGL	5.78E+04	4.22E+04	1.368	0.171	
RWB	1.49E+04	5.71E+03	2.606	**0.009**	
RUL	−2.58E+02	2.08E+02	−1.244	0.213	

*Note:* The full names of some variables were presented in the Methods. Statistically significant *p* values (*p* < 0.05) were indicated by bold font.

**TABLE 3:** Descriptive statistics of beta regression analyses when community metrics, function metrics, local‐scale environmental variables and regional land‐use variables were used as predictors of fish FLCBD.

	Estimate	SE	*z*	*p*	Pseudo‐*R* ^2^
Community metrics					
Intercept	−4.945	3.512	−1.408	0.159	0.187
Species richness	−5.682	6.807	−0.835	0.404	
Species richness^2^	2.425	3.685	0.658	0.510	
Community abundance	4.402	2.223	1.980	**0.048**	
Community abundance^2^	−1.028	0.584	−1.762	0.078	
Function metrics					
Intercept	−5.924	0.889	−6.661	**< 0.001**	0.783
Fdis	−3.722	0.720	−5.171	**< 0.001**	
Fric	0.072	0.442	0.163	0.871	
Fdiv	−0.546	0.709	−0.769	0.442	
Fori	−6.360	2.964	−2.146	**0.032**	
Fspe	13.060	1.743	7.492	**< 0.001**	
Fimb	0.043	0.333	0.130	0.897	
Local‐scale environmental variables					
Intercept	0.332	2.623	0.126	0.899	0.446
DO	−0.924	1.097	−0.842	0.400	
WT	**−3.864**	1.619	−2.387	**0.017**	
CO	0.078	0.461	0.168	0.866	
WW	0.243	0.387	0.628	0.530	
ED	0.609	2.252	0.270	0.787	
CV	2.398	2.522	0.951	0.342	
SC	**3.620**	1.159	3.125	**0.002**	
SH	−1.965	1.829	−1.074	0.283	
Land‐use variables					
Intercept	−3.286	0.217	−15.145	**< 0.001**	0.117
AL	−0.539	0.359	−1.502	0.133	
GL	−1.496	1.408	−1.062	0.288	
WB	−1.258	2.796	−0.450	0.653	
UL	0.986	2.074	0.475	0.634	
Intercept	−3.61E+00	1.51E−01	−23.901	**< 0.001**	0.166
RAL	−9.69E+00	3.03E+02	−0.032	0.975	
RFA	1.14E+03	1.06E+03	1.071	0.284	
RGL	1.02E+05	1.03E+05	0.985	0.325	
RWB	1.14E+04	1.31E+04	0.870	0.384	
RUL	−1.00E+03	4.39E+02	−2.283	**0.022**	

*Note:* The full names of some variables were presented in the Methods. Statistically significant *p* values (*p* < 0.05) were indicated by bold font.

**FIGURE 5:**
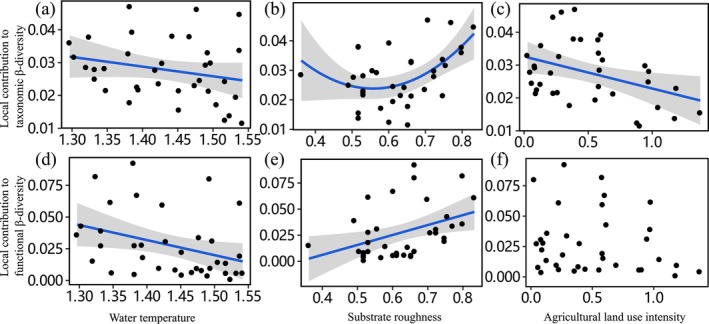
Relationships were fitted between LCBD values (taxonomy and function) and predictors (local environmental variables, and regional land use variables) separately. Shaded gray areas represent the confidence interval of 95% for the regression model. (a)‐(c) Represented the relationship between the local contribution to taxonomic β‐diversity and water temperature, substrate coarseness, and agricultural land use, respectively; (d)‐(f) Represented the relationship between the local contribution to functional β‐diversity and water temperature, substrate coarseness, and agricultural land use, respectively.

With respect to environmental drives, Beta regression analyses demonstrated that among the local environmental variables, both the TLCBD and FLCBD values were significantly negatively associated with water temperature (Tables 2 and 3; Figure 5a,d), and U‐shaped and linear positively related to substrate roughness, respectively (Tables 2 and 3; Figure 5b,e). For regional landscape variables, TLCBD values were significantly negatively related to agricultural land‐use intensity, and FLCBD values were significantly negatively associated with the change rate of urban land‐use intensity (Table 3). The relationships between TLCBD and FLCBD values and regional landscape variables support our third hypothesis, that is, stream sites affected by more intense anthropogenic land use have lower LCBD values.

### 3.3 Patterns of TSCBD and FSCBD and Their Drivers

The pattern of ecological uniqueness of fishes at the species level in the study area was as follows: TSCBD values ranged from 0.0003 to 0.1320, and the TSCBD values of nine species were greater than the average for all species; FSCBD values ranged from 0.0045 to 0.1379, and the FSCBD values of seven species were greater than the average for all species (Table S4). A moderate positive correlation between TSCBD and FSCBD values was found by Spearman correlation analysis (*r* = 0.607, *p* < 0.001; Figure 2b).

**FIGURE 6:**
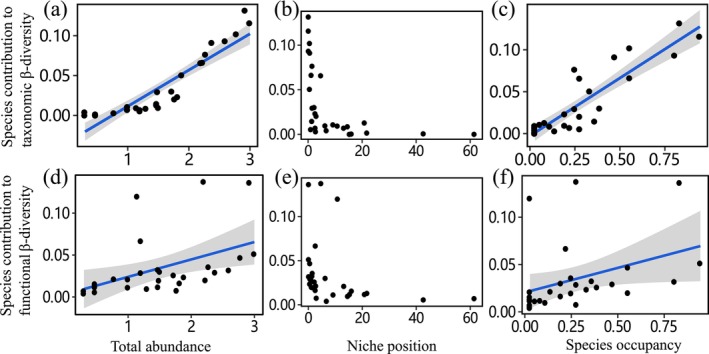
Linear relationships were fitted between SCBD values (taxonomy and function) and species feature separately. Only statistically significant (*p* < 0.05) relationships were showed in here. Shaded gray areas represent the confidence interval of 95% for the regression model. (a)‐(c) Represented the relationship between the species contribution to taxonomic β‐diversity and total abundance, niche position, and species occupancy, respectively; (d)‐(f) Represented the relationship between the local contribution to functional β‐diversity and total abundance, niche position, and species occupancy, respectively.

Our fourth hypothesis that SCBD values of fish communities were influenced by biological factors was confirmed. Specifically, Beta regression analyses revealed that both TSCBD and FSCBD values were significantly positively related to total species abundance and species occupancy, and negatively associated with niche position (Tables 4 and 5, Figure 6a–f).

**TABLE 4:** Descriptive statistics of beta regression analyses when species features (i.e., species occupancy and total abundance) and species niche (i.e., niche position and niche breadth) were used as predictors of fish TSCBD.

	Estimate	SE	*z*	*p*	Pseudo‐*R* ^2^
Intercept	−8.049	0.424	−19.006	**< 0.001**	0.907
Total abundance	3.426	0.418	8.204	**< 0.001**	
Total abundance^2^	−0.459	0.099	−4.634	**< 0.001**	
Intercept	−2.971	0.194	−15.338	**< 0.001**	0.508
Niche position	−0.057	0.013	−4.235	**< 0.001**	
Intercept	−3.472	0.280	−12.408	**< 0.001**	0.058
Niche breadth	0.061	0.066	0.929	0.353	
Intercept	−5.322	0.316	−16.872	**< 0.001**	0.707
Species occupancy	7.749	1.394	5.560	**< 0.001**	
Species occupancy^2^	−4.496	1.325	−3.394	**< 0.001**	
Intercept	−2.885	0.403	−7.165	**< 0.001**	0.077
Functional uniqueness	−3.786	2.894	−1.308	0.191	

*Note:* Statistically significant *p* values (*p* < 0.05) were indicated by bold font.

**TABLE 5:** Descriptive statistics of beta regression analyses when species features (i.e., species occupancy and total abundance), species niche (i.e., niche position and niche breadth) and functional traits were used as predictors of fish FSCBD.

	Estimate	SE	*z*	*p*	Pseudo‐*R* ^2^
Intercept	−4.088	0.509	−8.033	**< 0.001**	0.371
Total abundance	0.532	0.150	3.535	**< 0.001**	
Total abundance^2^	0.156	0.042	3.703	**< 0.001**	
Intercept	−3.133	0.165	−19.012	**< 0.001**	0.251
Niche position	−0.025	0.012	−2.154	**0.031**	
Intercept	−3.345	0.201	−16.660	**< 0.001**	0.002
Niche breadth	0.010	0.057	0.170	0.865	
Intercept	−3.819	0.278	−13.727	**< 0.001**	0.283
Species occupancy	1.334	0.434	3.076	**0.002**	
Species occupancy^2^	1.307	0.464	2.820	**0.005**	
Intercept	−3.211	0.329	−9.774	**< 0.001**	0.009
Functional uniqueness	−0.920	2.349	−0.392	0.695	

*Note:* Statistically significant *p* values (*p* < 0.05) were indicated by bold font.

## 4 Discussion

Assessing the relative contributions of local communities and single species to β‐diversity helps to better understand the dynamics of fish communities and their responses to anthropogenic disturbance. The findings of this study realized our initial research purposes. To be specific, the TLCBD value was hump‐shaped related to community abundance. For FLCBD values, it was positively associated with community abundance, functional originality, and functional specialization, and had a U‐shaped association with functional dispersion. In addition, both the TLCBD and FLCBD values were negatively associated with water temperature, and U‐shaped and linearly positively related to substrate roughness, respectively. For regional environmental variables, TLCBD values were negatively related to agricultural land‐use intensity, and FLCBD values were negatively associated with the change rate of urban land‐use intensity. Regarding SCBD values, both TSCBD and FSCBD values were positively related to total species abundance and species occupancy, and negatively associated with niche position. In summary, the results of this study indicated that LCBD values of stream fish were influenced by biological and environmental factors, and SCBD values were affected by biological factors. Meanwhile, the results also demonstrated that increasing disturbance reduces the fish's ecological uniqueness at the community level in the Xin'an River, that is, stream sites affected by more intense land use exhibit taxonomic and functional homogenization of fish communities. The possible mechanisms that led to these results are described below.

### 4.1 Different Drivers of TLCBD and FLCBD

The results verified our conjecture in this study, that is, the LCBD values of stream fish communities were influenced by biological and environmental factors. For instance, both the TLCBD and FLCBD values of the fish communities were correlated with community abundance, but their response patterns to changes in community abundance differed. There was a hump‐shaped relationship between TLCBD and community abundance; that is, the compositional uniqueness of local communities tended to increase and then decrease with increasing community abundance. According to previous studies, the relationship between the LCBD and community abundance depends to some extent on the species compositional characteristics of the community (Vilmi et al. 2017; de Bello et al. 2022). For example, Legendre and De Caceres (2013) reported that the LCBD values of streams with unique species compositions were negatively correlated with taxonomic α‐diversity. However, when there were more rare species in a species‐rich community, the LCBD values of local communities were positively related to species richness (Qiao et al. 2015; Kong et al. 2017). Thus, the relationship between LCBD values and community abundance was influenced by the relative abundance of common and rare species in the community (da Silva et al. 2018). In this study, TLCBD values were positively related to rare species abundance, indicating that within a certain threshold of community abundance, rare species are the dominant factor in compositional uniqueness. In contrast, when community abundance exceeds a certain threshold, common species replace rare species as the dominant factor in compositional uniqueness. In the context of this study, sampling sites with rare species, such as *L. taeniops* , *Saurogobio dabryi* , *Sinobdella sinensis* , and *Sarcocheilichthys nigripinnis* , generally had higher TLCBD values than the mean values of the study area (Table S2). In contrast to TLCBD, the FLCBD values were linearly positively associated with community abundance, which can be explained by rare species. This is because we found that only the FLCBD values were linearly positively related to the abundance of rare species. The sampling sites where rare species such as *R. oxycephalus* , *S. nigripinnis* , and *O. sinensis* occur usually have higher FLCBD values than the mean values of the study area (Table S2). No relationship was found between the abundance of common or occasional species and FLCBD values, which may be due to the high degree of functional trait redundancy among common and occasional species (Tan 2022). The results demonstrated that changes in LCBD values (whether related to a taxon or a function) in the Xin'an River were impacted by changes in community abundance, further emphasizing that sites with higher LCBD values should be prioritized in conservation plans (Legendre and De Caceres 2013; da Silva et al. 2018; Leão et al. 2020).

In addition to community abundance, functional α‐diversity also has a complex relationship with FLCBD values (da Silva et al. 2020; Xia et al. 2022). Among the six functional α‐diversity indices, only functional dispersion, functional originality, and functional specialization were significantly correlated with FLCBD values. The curves illustrated that, compared with less unique communities, groups that are highly unique in terms of species composition are likely to lack some species with rare traits. However, this loss of function does not imply the loss of some specialized and original functions performed by fish because high functional specialization and originality denote a species‐specific combination of traits (Mouillot et al. 2008). No relationships were found between functional richness, functional divergence, and FLCBD values, indicating that there were no dramatic changes in niche use or niche complementarity in local communities, regardless of high or low functional specificity. FLCBD values were not related to functional imbalance, further supporting our speculation. This suggests an imbalance in the distribution of species abundance in functional space (Ricotta et al. 2022; Xia et al. 2022). The above results may be related to the regular distribution of functional traits in functional space, relatively loose niche space, and low competition among species (Zhang et al. 2019). Our results are similar to those found by Xia et al. (2022) in the Chishui River. However, many studies have also shown that the FLCBD values of the dung beetle community were negatively correlated with multifaceted functional diversity, suggesting that the relationship between FLCBD values and the functional α‐diversity index may be specific to each animal group (da Silva et al. 2020; Paquette et al. 2021; Santos et al. 2021; Xia et al. 2022).

The results also confirmed our conjecture that the LCBD values of stream fish communities were influenced by environmental factors in this study. The LCBD values of the fish communities were related to regional scale environmental variables. For example, the area of agricultural land use and the rate of urban land‐use change had negative effects on the TLCBD and FLCBD values, respectively, and the effects of land use were more pronounced on the TLCBD values than on the FLCBD values. Empirical evidence suggests that both less degraded (Heino et al. 2017) and more degraded (Leão et al. 2020; Bomfim et al. 2023) sampling sites may have a high degree of compositional uniqueness along the land‐use gradient. We found that compositional uniqueness was greater at the less degraded sampling sites in this study. This may be related to the greater number of rare species in the less degraded sites. For example, sampling sites with rare species such as *Onychostoma barbatulum*, *L. taeniops* , and *R. oxycephalus* had lower levels of disturbance. This finding is consistent with our earlier finding that the number of rare species is positively correlated with TLCBD and FLCBD values. However, we found that the compositional uniqueness of fish communities responded differently to disturbance gradients than that of other taxa (e.g., aquatic insects and diatoms; de Paiva et al. 2021; Schneck et al. 2022; Bomfim et al. 2023). This may be due to the difficulty in identifying the most important predictors of LCBD values and the fact that biological populations respond differently to different predictors (Landeiro et al. 2018). The LCBD values of the fish communities were also related to local‐scale environmental variables. As we know, anthropogenic disturbances (e.g., deforestation, dams, and agriculture) generally affect stream fish diversity by affecting the local environment (Zhang et al. 2019; Camana et al. 2020; Brejão et al. 2021; Qiao et al. 2022). Here, we found that both current land use and land‐use change experienced over 30 years affect the local environment of headwater streams to varying degrees (Qiao 2023). Highly specialized local environments select species with similar functional traits through environmental filtering, which ultimately changes the species composition of local communities and increases the β‐diversity between communities in the region (Leibold et al. 2004; Pajunen et al. 2017; Leão et al. 2020). Among the many environmental factors, water temperature is one of the most important in controlling the distribution and abundance of freshwater fish (Matthews 2012). It directly affects their metabolism, reproduction, development, growth, and behavior (Jackson et al. 2001). We found that water temperature was negatively correlated with TLCBD and FLCBD values in this study. Substrate roughness had a U‐shaped relationship with TLCBD values and a positive relationship with FLCBD values. Substrate composition is important for ecological processes such as spawning, reproduction, and predation avoidance, which in turn affect fish distribution and abundance (e.g., *Cobitis rarus* and *V. stenosoma* preferred rock crevices; *Pseudorasbora parva* and *Carassius auratus* preferred silt environments). Regarding the effects of land‐use intensity, water temperature, and substrate roughness on LCBD values, the results also suggested that with the increasing of land‐use intensity or/and rising of temperatures (e.g., global warming, urbanization, and deforestation) or/and decreasing of substrate roughness, natural communities may experience exacerbated taxonomic and functional homogenization in the study area (Qiao et al. 2024). In addition, some studies have reported that environmental variables such as water width, water depth, and dissolved oxygen influence the ecological uniqueness of fish communities to varying degrees (Tonkin et al. 2016; Xia et al. 2022). However, this study did not detect relationships between these environmental variables and TLCBD or FLCBD, which may be related to the sampling setting in the study area. Although the sampling sites were located within the land‐use gradient, they were located in a single watershed with a single anthropogenic disturbance, a low degree of degradation, and low changes in some environmental variables between streams (de Paiva et al. 2021). Nevertheless, our findings are inconsistent with those of Heino and Gronroos (2017), who reported that the LCBD values of stream insects were not well explained by environmental variables, while our results reinforce the idea that the associations between LCBD values and environmental variables (regional and local environment) depend on the environment and the need to explore the relationship between ecological uniqueness and environmental variables.

### 4.2 Different Drivers of TSCBD and FSCBD

Since the relative contribution of species to β‐diversity can be used to identify key species that sustain changes in community composition within a given region, many scholars have advocated exploring SCBD values and associated biological factors (e.g., community attributes and functional traits) to better understand the drivers of β‐diversity patterns (Heino and Gronroos 2017; da Silva et al. 2018; Schneck et al. 2022). The results showed that there was a moderate correlation between TLCBD and FSCBD values; however, there were some differences in the species composition of communities with higher TLCBD and FSCBD values. The above results may be due to the possible functional redundancy of species between local communities in the study area, as the greater the functional redundancy of species between local communities is, the more obvious the differences in regional taxonomic and functional β‐diversity are, which may lead to differences in species contributions to taxonomic and functional β‐diversity (Gianuca et al. 2018). It has also been shown that multidimensional community diversity needs to be considered when assessing species contributions to fish community β‐diversity (Nakamura et al. 2020; Zhao et al. 2021; Tan 2022).

In this study, the results also realized our aim that the SCBD values of fish communities were influenced by biological factors. For instance, SCBD values were found to be positively correlated with total species abundance and species occupancy, such a phenomenon that has also been reported in other aquatic taxa (e.g., aquatic insects and diatoms; Heino and Gronroos 2017; Vilmi et al. 2017; de Paiva et al. 2021). This suggests that the species that contributed most to the β‐diversity in this study were more abundant in individuals and occupied sites with different levels of environmental gradients. Given this, if species abundance varies considerably between different sampling sites, it can have considerable effects on β‐diversity (Vilmi et al. 2017; de Paiva et al. 2021). In this study, the species that contributed most to the TSCBD and FSCBD values included *Z. platypus* , *Rhinogobius* spp., and *V. stenosoma* (Table S4), which were relatively abundant (14.87% to 24.65%) and widely distributed (55.56% to 94.44%) (Table S4). The above results are also applicable to other freshwater and terrestrial ecosystems (Siqueira et al. 2009; da Silva et al. 2018; Xia et al. 2022). In addition, niche position was negatively correlated with SCBD values, which is consistent with the findings of previous studies (Heino and Gronroos 2017; da Silva et al. 2018; Xia et al. 2022). As niches are generally negatively correlated with species occupancy, it can be inferred that niche position is correlated with SCBD values calculated based on abundance data (Heino and Tolonen 2017). Compared to nonmarginal species (e.g., *Z. platypus* and *V. stenosoma* ), marginal species (e.g., *L. taeniops* , *O*. *barbatulum*, and *S. dabryi* ) that occur only in certain types of habitat patches contribute less to β‐diversity. Environmental variables can explain the extent of species occupancy, which is mainly driven by niche (Heino and de Mendoza 2016). However, our results do not fully support the notion that niche breadth is an important factor influencing SCBD values, which is consistent with the findings of some studies (Tan 2022; Xia et al. 2022). It is possible that niche breadth is not critical in the management of SCBD (da Silva et al. 2018; Leão et al. 2020).

## 5 Conclusions

We have decomposed β‐diversity into LCBD and SCBD, and explored their spatial distribution patterns and biological and environmental drivers in this study. Our study suggests that conservation efforts should consider both LCBD and SCBD in context and emphasize the importance of protecting headwater streams. Our main conclusions are as follows: The conversion of natural landscapes into artificial landscapes around streams has reduced the ecological uniqueness (TLCBD and FLCBD) of fish communities in the headwater stream of the Xin'an River, where habitat variables such as water temperature and substrate had important effects on ecological uniqueness (TLCBD and FLCBD). In other words, human‐induced land conversion and local environmental changes promote taxonomic and functional homogenization of fish communities in the studied area. Furthermore, sampling sites with greater ecological uniqueness at the taxonomic level also had greater ecological uniqueness at the functional level; specifically, sampling sites with greater community abundance may have greater functional specialization and functional originality. Thus, the joint conservation of sampling sites with high LCBD values and high community abundances can not only protect a greater proportion of regional species diversity but also functional diversity, which is important for maintaining ecosystem function. SCBD values were associated with species occupancy, abundance, and niche position. We suggest that consideration of SCBD as part of a conservation approach needs to be coupled with species abundance and occupancy, which will help to more comprehensively prioritize areas for conservation and restoration.

## Author Contributions

**Jialing Qiao:** conceptualization (lead), data curation (lead), formal analysis (lead), methodology (lead), software (lead), writing – original draft (lead), writing – review and editing (lead). **Ling Chu:** conceptualization (supporting), data curation (lead), formal analysis (lead), investigation (lead), software (lead), writing – original draft (equal). **Yuru Li:** writing – original draft (supporting), writing – review and editing (supporting). **Tianjiang Chu:** writing – review and editing (supporting). **Nan Xie:** project administration (lead), resources (lead), supervision (lead), writing – review and editing (equal). **Yunzhi Yan:** investigation (lead), project administration (lead), resources (lead), supervision (lead), writing – review and editing (equal).

## Conflicts of Interest

The authors declare no conflicts of interest.

## Supporting information


Table S1.

## Data Availability

The data used to support the findings of this study are available on the Data Dryad Digital Repository, https://datadryad.org/stash/share/zY‐‐CG_XdCtVa0TY_AqE1SFcOIeC_tzPkmnUKqNb31s.
