# Supplementary material for: Unraveling Spatial Patterns and Drivers of Fish Ecological Uniqueness in Subtropical Streams
Source: Ecol Evol. 2025 Mar 27;15(4):e71112. doi: 10.1002/ece3.71112 (PMC11949569; doi:10.1002/ece3.71112)
Supplement: Supplementary file 1 — Table S1. [file ECE3-15-e71112-s001.docx]

**Table S1** Species composition, occurrence of frequency (FO) and relative abundance (RA) of fishes in the Xin’an River.

| Order/family/species | Occurrence of frequency (FO%) | Relative abundance (RA%) |
| --- | --- | --- |
| **Cypriniformes** |  |  |
| **Cobitidae** |  |  |
| *Misgurnus anguillicaudatus* | 55.56 | 4.15 |
| *Parabotia fasciata* | 25.00 | 0.74 |
| *Cobitis sinensis* | 36.11 | 0.72 |
| *Cobitis rarus* | 47.22 | 5.90 |
| *Leptobotia taeniops* | 2.78 | 0.05 |
| **Homalopteridae** |  |  |
| *Vanmanenia stenosoma* | 55.56 | 14.87 |
| **Cyprinidae** |  |  |
| *Acrossocheilus fasciatus* | 80.56 | 9.76 |
| *Zacco platypus* | 94.44 | 24.65 |
| *Opsarrichthys bidens* | 27.78 | 1.43 |
| *Rhynchocypris oxycephalus* | 5.56 | 0.22 |
| *Rhodeus ocellatus* | 27.78 | 3.88 |
| *Pseudorasbora parva* | 19.44 | 0.77 |
| *Sarcocheilichthys parvus* | 25.00 | 4.64 |
| *Abbottina rivularis* | 19.44 | 1.61 |
| *Squalidus argentatus* | 33.33 | 1.88 |
| *Gnathopogon imberbis* | 2.78 | 0.02 |
| *Saurogobio dabryi* | 2.78 | 0.05 |
| *Carassius auratus* | 22.22 | 0.37 |
| *Microphysogobio fukiensis* | 11.11 | 0.47 |
| *Onychostoma barbatulum* | 2.78 | 0.05 |
| *Acheilognathus gracilis* | 2.78 | 0.32 |
| *Sarcocheilichthys nigripinnis* | 8.33 | 0.72 |
| **Siluriformes** |  |  |
| **Bagridae** |  |  |
| *Pseudobagrus truncatus* | 27.78 | 0.37 |
| **Symbranchiformes** |  |  |
| **Symbranchidae** |  |  |
| *Monopterus albus* | 13.89 | 0.12 |
| **Mastacembelidae** |  |  |
| *Sinobdella sinensis* | 2.78 | 0.05 |
| **Perciformes** |  |  |
| **Odontobutidae** |  |  |
| *Odontobutis potamophila* | 38.89 | 1.28 |
| **Eleotridae** |  |  |
| *Hypseleotris swinhonis* | 2.78 | 0.02 |
| **Gobiidae** |  |  |
| *Rhinogobius* spp. | 83.33 | 20.67 |
| **Beloniformes** |  |  |
| **Adrianichthyidae** |  |  |
| *Oryzias sinensis* | 2.78 | 0.22 |

**Table S2** TLCBD and FLCBD values in 36 sites of the Xin'an River.

| sites | TLCBD-value | FLCBD-value |
| --- | --- | --- |
| site1 | 0.03802047 | 0.006572047 |
| site2 | 0.01392287 | 0.001174817 |
| site3 | 0.01256218 | 0.005947042 |
| site4 | 0.0195817 | 0.005985327 |
| site5 | 0.02311879 | 0.009735705 |
| site6 | 0.03000433 | 0.03137953 |
| site7 | 0.01728923 | 0.014587082 |
| site8 | 0.02366371 | 0.059759907 |
| site9 | 0.01566411 | 0.004518003 |
| site10 | 0.0381621 | 0.008587697 |
| site11 | 0.04479888 | 0.061265785 |
| site12 | 0.01166218 | 0.006064735 |
| site13 | 0.03384231 | 0.019507434 |
| site14 | 0.02141792 | 0.009856384 |
| site15 | 0.02160665 | 0.006268113 |
| site16 | 0.01790459 | 0.005045611 |
| site17 | 0.0471624 | 0.027718505 |
| site18 | 0.02182274 | 0.018352339 |
| site19 | 0.03280617 | 0.092721421 |
| site20 | 0.03178111 | 0.043151916 |
| site21 | 0.0282869 | 0.061848802 |
| site22 | 0.03939027 | 0.067401101 |
| site23 | 0.0378915 | 0.082180761 |
| site24 | 0.02865278 | 0.015555232 |
| site25 | 0.02509737 | 0.039328933 |
| site26 | 0.02989626 | 0.030932115 |
| site27 | 0.02435502 | 0.013803482 |
| site28 | 0.03312575 | 0.080386724 |
| site29 | 0.02153718 | 0.022520238 |
| site30 | 0.02264914 | 0.028153646 |
| site31 | 0.02936082 | 0.003883736 |
| site32 | 0.04632887 | 0.034054156 |
| site33 | 0.02779567 | 0.009867351 |
| site34 | 0.02467413 | 0.008067568 |
| site35 | 0.0280281 | 0.027604657 |
| site36 | 0.0361358 | 0.036212096 |

**Table S3** Descriptive statistics of beta regression analyses when the abundance of common species, accidental species and rare species were used as predictors of fish TLCBD and FLCBD values. Statistically significant p values (p < 0.05) were indicated by bold font.

|  |  | Estimate | Std. Error | z-value | Pr(>\|z\|) | Pseudo R^2^ |
| --- | --- | --- | --- | --- | --- | --- |
| TLCBD | (Intercept) | -3.071 | 0.164 | -18.697 | **<0.001** | 0.1961 |
|  | Common species | -0.295 | 0.097 | -3.038 | **0.002** |  |
|  | (Intercept) | -3.598 | 0.103 | -35.060 | **<0.001** | 0.0064 |
|  | Accidental species | 0.045 | 0.089 | 0.502 | 0.615 |  |
|  | (Intercept) | -3.624 | 0.062 | -58.763 | **<0.001** | 0.1172 |
|  | Rare species | 0.308 | 0.123 | 2.502 | **0.0123** |  |
| FLCBD | (Intercept) | -3.593 | 0.436 | -8.247 | **<0.001** | 0.0003 |
|  | Common species | 0.022 | 0.242 | 0.092 | 0.927 |  |
|  | (Intercept) | -3.628 | 0.246 | -14.750 | **<0.001** | 0.0046 |
|  | Accidental species | 0.076 | 0.200 | 0.380 | 0.704 |  |
|  | (Intercept) | -3.812 | 0.161 | -23.632 | **<0.001** | 0.1676 |
|  | Rare species | 0.922 | 0.246 | 3.747 | **<0.001** |  |

**Table S4** TSCBD and FSCBD values in 29 species of the Xin'an River.

| species | TSCBD-value | FSCBD-value |
| --- | --- | --- |
| *Abbottina rivularis* | 0.023500774 | 0.016677446 |
| *Microphysogobio fukiensis* | 0.008892384 | 0.009936091 |
| *Rhodeus ocellatus* | 0.066093967 | 0.137780423 |
| *Acrossocheilus fasciatus* | 0.093595192 | 0.032156602 |
| *Odontobutis potamophila* | 0.030441065 | 0.026164865 |
| *Gnathopogon imberbis* | 0.004445826 | 0.004386201 |
| *Sarcocheilichthys nigripinnis* | 0.013140263 | 0.012078097 |
| *Parabotia fasciata* | 0.029741231 | 0.019865119 |
| *Monopterus albus* | 0.003267059 | 0.021660142 |
| *Hypseleotris swinhonis* | 0.000404166 | 0.00737995 |
| *Carassius auratus* | 0.007281699 | 0.066995549 |
| *Rhynchocypris oxycephalus* | 0.011035976 | 0.01154884 |
| *Zacco platypus* | 0.116187425 | 0.051596961 |
| *Opsarrichthys bidens* | 0.020616729 | 0.007855282 |
| *Pseudorasbora parva* | 0.009892127 | 0.030327586 |
| *Misgurnus anguillicaudatus* | 0.066591338 | 0.020258899 |
| *Pseudobagrus truncatus* | 0.005691108 | 0.028971886 |
| *Saurogobio dabryi* | 0.00096126 | 0.005954675 |
| *Onychostoma barbatulum* | 0.000340894 | 0.011954356 |
| *Rhinogobius* spp. | 0.132041436 | 0.136648344 |
| *Acheilognathus gracilis* | 0.009632623 | 0.120181929 |
| *Cobitis rarus* | 0.091544095 | 0.02947977 |
| *Sarcocheilichthys parvus* | 0.076767549 | 0.036014006 |
| *Squalidus argentatus* | 0.050841188 | 0.023832343 |
| *Vanmanenia stenosoma* | 0.102332246 | 0.047070537 |
| *Sinobdella sinensis* | 0.001839652 | 0.013341914 |
| *Cobitis sinensis* | 0.014889637 | 0.032738204 |
| *Oryzias sinensis* | 0.007274988 | 0.021282879 |
| *Leptobotia taeniops* | 0.000716106 | 0.015861105 |
